# Magnetic Resonance Imaging of Primary Adult Brain Tumors: State of the Art and Future Perspectives

**DOI:** 10.3390/biomedicines11020364

**Published:** 2023-01-26

**Authors:** Matia Martucci, Rosellina Russo, Francesco Schimperna, Gabriella D’Apolito, Marco Panfili, Alessandro Grimaldi, Alessandro Perna, Andrea Maurizio Ferranti, Giuseppe Varcasia, Carolina Giordano, Simona Gaudino

**Affiliations:** 1 Dipartimento di Diagnostica per Immagini, Radioterapia Oncologica ed Ematologia, Fondazione Policlinico “A. Gemelli” IRCCS, 00168 Rome, Italy; 2 Istituto di Radiologia, Università Cattolica del Sacro Cuore, 00168 Rome, Italy

**Keywords:** brain tumor imaging, MRI, advanced MR Imaging, perfusion MRI, functional MRI, DTI, MR spectroscopy, AI, quantitative MRI

## Abstract

MRI is undoubtedly the cornerstone of brain tumor imaging, playing a key role in all phases of patient management, starting from diagnosis, through therapy planning, to treatment response and/or recurrence assessment. Currently, neuroimaging can describe morphologic and non-morphologic (functional, hemodynamic, metabolic, cellular, microstructural, and sometimes even genetic) characteristics of brain tumors, greatly contributing to diagnosis and follow-up. Knowing the technical aspects, strength and limits of each MR technique is crucial to correctly interpret MR brain studies and to address clinicians to the best treatment strategy. This article aimed to provide an overview of neuroimaging in the assessment of adult primary brain tumors. We started from the basilar role of conventional/morphological MR sequences, then analyzed, one by one, the non-morphological techniques, and finally highlighted future perspectives, such as radiomics and artificial intelligence.

## 1. Introduction

Brain tumors (BTs) are a significant burden on people’s health and on public healthcare, due to the poor prognosis of malignant subtypes (average five-year survival of 35%). Worldwide, 308,102 new cases of primary brain and CNS cancers were diagnosed, and 251,329 people died from these malignancies in 2020 [1]. In the era of precision medicine, an early diagnosis and an accurate follow-up are needed for better patient care. In this scenario, magnetic resonance imaging (MRI) significantly contributes to diagnosis and plays a key role in therapy planning and in evaluating treatment response and/or recurrence.

The evolution of MR equipment and techniques has gone hand in hand with the ever-increasing needs of clinicians (surgeons, oncologists, radiotherapists) [2], so that the current state of neuroimaging has evolved into a comprehensive diagnostic tool that allows for the characterization of morphologic as well as functional, hemodynamic, metabolic, cellular, microstructural, and sometimes even genetic information of BTs.

The purpose of this article was to provide a comprehensive overview about the role of neuroimaging in the assessment of adult primary brain tumors. We started by discussing morphological MR sequences, then analyzed, one by one, the non-morphological techniques, providing a brief overview on the technical aspects followed by a more practical description of their clinical application. Finally, we presented an outline of future perspectives, such as radiomics and artificial intelligence.

## 2. Conventional/Morphological MRI

MRI is the leading imaging modality in patients with primary intra-axial BTs [2]. Computed tomography (CT) has limited indications, mainly in emergency settings (detection of intratumoral acute hemorrhage or critical mass effect as cerebral herniation or hydrocephalus) or, less frequently, in detection/confirmation of calcifications. A standard MRI protocol for BTs should always include T2 weighted (T2w) sequences in at least two orthogonal planes (i.e., axial and coronal), 2D or preferably (if available) 3D-FLAIR (fluid-attenuated inversion recovery), high-resolution 3D T2* gradient echo sequences as susceptibility weighted imaging (SWI) [3], axial pre- and post-contrast T1 weighted sequences, the latter preferably with 3D high-resolution iso-volumetric sequences (such as spoiled gradient recalled acquisition or similar), which are useful in pre-treatment workup for intraoperative MR guided navigational system or for stereotactic radiotherapy (RT) planning [4,5].

The main roles of conventional/morphological MRI at the diagnosis are to determine the size and the anatomical location of the lesion within the brain for treatment or biopsy planning, to evaluate mass effect and edema in surrounding healthy brain tissue, to assess the relationship with ventricular system and brain vascular structures, and finally, along with other “functional” MRI sequences, to suggest a possible diagnosis [2].

The Publication of the 2021 World Health Organization (WHO) Classification of Tumors of the Central Nervous System (CNS) has revolutionized the diagnostic workup of CNS neoplasms, making the diagnosis of a specific tumor type particularly challenging. In fact, the WHO 2021 classification has incorporated both histologic features and genetic alterations into the diagnostic framework, with fundamental prognostic and therapeutical implications [6,7]. Nonetheless, a detailed and comprehensive analysis of conventional/morphological and ‘functional’ MR features can actually lead to a suggested shortlist of likely possibilities.

**Figure 1:**
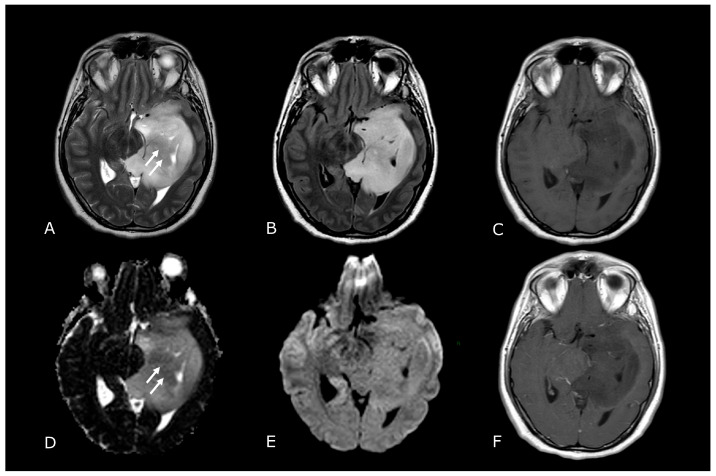
Morphological sequences: tumor cellularity. Morphological sequences: tumor cellularity. Left deep temporo-mesial WHO grade 3 IDH-mut diffuse astrocytoma. Lesion is hyperintense on T2w and FLAIR images (**A**,**B**), isointense on DWI (**E**), with areas of lower T2 signal intensity and diffusion restriction (white arrows in (**A**,**D**) that reflect hypercellular and probably more anaplastic tissue. No contrast enhancement is detectable on post-contrast T1w image (**F**) compared to pre-contrast image (**C**).

**Figure 2:**
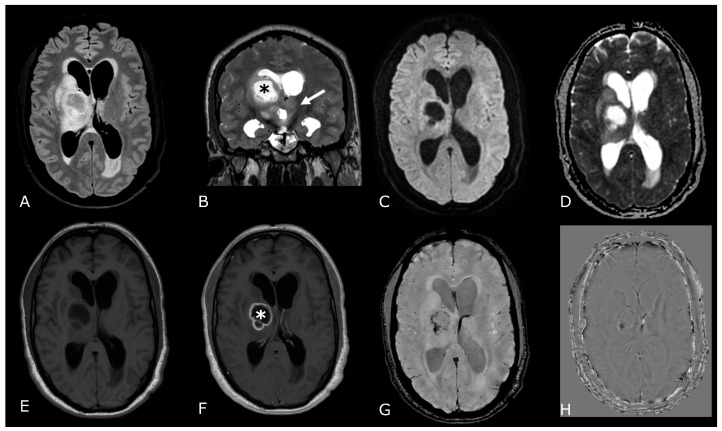
Conventional MR sequences: necrosis and hemorrhages. Right deep thalamo-capsular IDH-wt GB. The lesion shows a necrotic core (asterisk in (**B**,**F**)) and a thick and irregular rim of hypercellular tissue with restricted diffusion (**C**,**D**) and contrast-enhancement ((**F**) compared to (**E**)). (**G**,**H**) demonstrate hemosiderin marginal deposits (hypointense on both SWI and phase-map respectively) suggestive of intratumoral bleedings. The “rim enhancing” lesion is surrounded by a peripheral heterogeneous area of abnormal T2w/FLAIR signal (**A**,**B**), reflecting infiltrative “non enhancing” tumor and vasogenic edema that also involves the mesial surface of the contralateral thalamus and hypothalamus (arrow in (**B**). The caudal extension determines stenosis of the Sylvian aqueduct and consequently supratentorial hydrocephalus.

From a general point of view, T2w/FLAIR images generally represent the key to identify any ‘brain lesion’, of any nature/origin. With regard to BTs, these sequences allow the detection of the lesion and in some way to tumor characterization. In fact, the T2w signal may reflect tissue density and thus tumor cellularity: the more cellularity, the lower the T2w signal (Figure 1). However, the T2w/FLAIR hyperintense signal does not always signify a tumor. Signal abnormality may represent peritumoral edema or, as with infiltrative gliomas, both edema and infiltrative tumor tissue [8]. Moreover, as many tumors have overlapping T2w/FLAIR imaging findings, knowing other potentially distinguishing features (such as calcification, hemorrhages, or necrosis) is useful to limit differential diagnoses (Figure 2).

SWI can help differentiate calcification from hemorrhages because calcification is diamagnetic, whereas most hemorrhagic byproducts are paramagnetic. Due to their opposite magnetic susceptibilities, their phase deflections are opposite as well [9]. Dense coarse calcifications are seen in 34–80% of oligodendrogliomas (ODs) [10], making them the intra-axial tumors with the highest frequency of calcification among BTs. Although the likelihood of an OD is high for a calcified supratentorial intraparenchymal tumor, the differential diagnosis of a calcified intra-axial intracranial mass includes other tumors, such as ependymomas and low-grade astrocytomas.

Cystic components are more frequently associated with lower grade gliomas (e.g., pilocytic astrocytoma). On the contrary, intra-tumoral necrotic areas are mostly seen in higher grade tumors and depicted as areas of lack of enhancement on post gadolinium-based contrast agent (GBCA) T1w images (Figure 2F) [11].

Ultimately, intratumoral contrast enhancement is generally considered to be associated with higher tumor grade, although certain low-grade gliomas (LGG), such as pilocytic astrocytomas, generally enhance and certain high-grade gliomas (HGG) may not (Figure 1F) [12].

Going back to the WHO 2021 classification, adult-type diffuse gliomas are divided into:

- Astrocytoma, IDH-mutant (IDH-mut);
- Oligodendroglioma, IDH-mut and 1p/19q-codeleted;
- Glioblastoma (GB), IDH-wildtype (IDH-wt).

Additionally, IDH-mut diffuse astrocytomas are now graded 2–4 within type and the terms IDH-mut “anaplastic astrocytoma” and “glioblastoma” have been dropped.

In this new scenario, in which histopathology is integrated with tumor genetics for the classification of brain tumors, some “morphological” MRI features have demonstrated a significant correlation not only with histologic grade, but also with the mutational status of adult gliomas.

**Figure 3:**
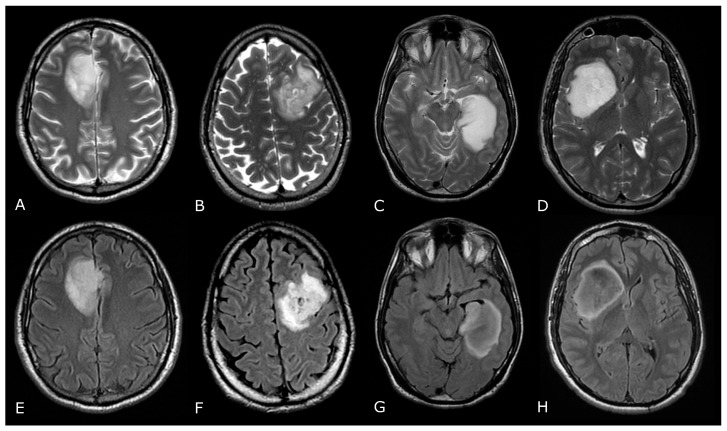
T2/FLAIR Mismatch Sign. Upper Row Ax T2w images, Lower Row Ax T2-FLAIR images. (**A**,**E**); (**B**,**F**) IDH-mut, 1p-19q codeleted ODs, respectively grade 2 (**A**,**E**) and grade 3 (**B**,**F**). (**C**,**G**); (**D**,**H**) IDH-mut, 1p-19q non codeleted Diffuse Astrocitomas, respectively grade 2 (**C**,**G**) and grade 3 (**D**,**H**). The T2-FLAIR mismatch sign (**C**) vs. (**G**) and (**D**) vs. (**H**) represents the T2 signal homogeneity of the mass with relatively hypointense signal throughout most of the lesion on FLAIR except for a peripheral rim of hyperintense signal. Notably, imaging features of grade 3 IDH-mut diffuse astrocytoma and OD may be indistinguishable from grade 2 IDH-mut diffuse astrocytoma and OD. However, grade 3 astrocytoma and OD may have more T2 signal heterogeneity (**B**,**D**).

Grade 2 IDH-mut diffuse astrocytoma frequently appears as a homogeneous T2-hyperintense circumscribed supratentorial mass, more typically located in the frontal or temporal lobes, without calcification or enhancement. Moreover, IDH-mut diffuse astrocytomas are characterized by the recently described “T2-FLAIR mismatch sign” (Figure 3), which consists of T2 homogeneity, with a relatively FLAIR hypointense signal throughout most of the lesion except for a peripheral rim of hyperintense signal. This sign has demonstrated high specificity but low sensitivity [13].

Grade 3 IDH-mut diffuse astrocytoma may be morphologically indistinguishable from grade 2, but the former tends to have greater T2 heterogeneity and post-contrast enhancement [14].

IDH-mut and 1p/19q-codeleted ODs typically involve frontal lobes, display intratumoral heterogeneity and calcification, with a variable degree of enhancement.

Finally, the presence of any of the following five histologic and molecular criteria is sufficient to designate an IDH-wt diffuse astrocytic glioma as a GB (grade 4 by definition): microvascular proliferation, necrosis, telomerase reverse transcriptase promoter (TERTp) mutation, epidermal growth factor receptor (EGFR) gene amplification, and +7/−10 chromosome copy number changes. At MRI, GBs typically present more aggressive and heterogenous morphological features: larger lesions, relevant mass effect and surrounding edema, intratumoral hemorrhage and necrosis, thick ring or irregular/multinodular contrast enhancement (Figure 2).

Although MRI can suggest possible diagnoses, histopathology and molecular analyses are necessary to provide definitive and accurate diagnoses.

As said above, morphological MRI plays a fundamental role even in the post-treatment follow-up. Interpreting findings after surgery, radiation, and chemotherapy requires profound knowledge of tumor biology, as well as of the peculiar changes expected to ensue as a consequence of each treatment technique.

The Response Assessment in Neuro-Oncology (RANO) criteria were developed as an objective tool for radiologic assessment of treatment response in high-grade gliomas. The updated RANO working group defines a complete response, partial response, stable disease or progressive disease to therapy for high-grade gliomas as listed in Table 1.

These assessment criteria provide a framework for treatment response evaluation but do not account for some not univocal post-treatment brain modifications such as pseudo-response (PsR) or pseudo-progression (PsP). A PsR may be seen with a marked decrease in tumor contrast enhancement following treatment with antiangiogenic agent (as bevacizumab) [16]. On the other hand, PsP may be seen as an increase in the tumor contrast-enhancing portion and an increase of the T2w/FLAIR surrounding signal abnormality after RT alone or with temozolomide. PsP is seen in around 20% of patients (approximately 50% of the patients who initially “progress” on imaging) [17]. The detailed discussion of these entities is beyond the purpose of this paper.

## 3. MR Perfusion

### 3.1. Overview and Techniques

Perfusion weighted imaging (PWI) denotes a variety of MRI techniques able to give insights into tissues’ blood perfusion, by using an intravascular tracer which can be detected in the area of interest. In brain PWI, it is possible either to use an exogenous, intravascular, contrast agent such as GBCA, or to use the patient’s own water molecules as an endogenous tracer [18].

Brain PWI performed after intravenous administration of GBCA analyzes quantitative cerebral perfusion parameters by exploiting the signal changes through a series of dynamic images following bolus administration and falls into two categories: dynamic susceptibility-weighted contrast (DSC); and dynamic contrast enhancement (DCE).

On the other hand, PWI without the use of GBCA, called arterial spin labeling (ASL), is performed through “spin tagging”, which means that diffusible and magnetically labeled protons within blood and water flow into the brain, where they are imaged.

Depending on the clinical-neuroradiological scenario, qualitative, semi-quantitative and quantitative approaches, such as the review of color-coded maps to the region of interest (ROI) analysis and analysis of signal intensity curves, can be applied [18].

### 3.2. Clinical Applications

DSC-PWI is the most used and validated perfusion imaging technique, particularly dealing with brain tumors [19]. The relative cerebral blood volume (rCBV) and the percentage of signal intensity recovery (PSR) derived from DSC-PWI provide information about neoangiogenesis and tumor capillary permeability respectively, which can be used to characterize tumor type and grade, and to distinguish tumor recurrence from post-treatment changes.

In recent years, DCE-MRI is increasingly being used for a more accurate mapping of blood volumes in tumor lesions, and because it can add useful information for tumor grading, differential diagnosis, evaluation of RT treatment effect, or for chemotherapy treatment response monitoring. Some authors strongly underline how this technique can represent a better alternative to DSC, given the drawback of the DSC to surgery- and blood-dependent artifacts [20,21]. The main parameters indicating increased tumor vascularity and BBB permeability are the area under contrast curve (AUC) and the Ktrans respectively. Other DCE-derived parameters are the Ve and Vp, which represent the fractional volume of the GBCA in the extravascular-extracellular space and in the plasma space respectively. Longer scanning time, reduced temporal resolution, complex post-processing and quantification of images are the disadvantages of DCE, which make it less often used in clinical practice and less frequently investigated in literature compared to DSC [22].

Regarding ASL, although completely noninvasive and potentially repeatable as many times as necessary, it is still rarely used in neuro-oncology, mostly because the standard MRI protocol for BTs requires the administration of GBCA, thus making it preferable to use faster and more robust/validated techniques such as DSC and/or DCE, instead of ASL.

The main parameters derived from brain perfusion techniques are summarized in Table 2.

#### 3.2.1. Differentiating HGG from LGG and Clinical Prognosis

**Figure 4:**
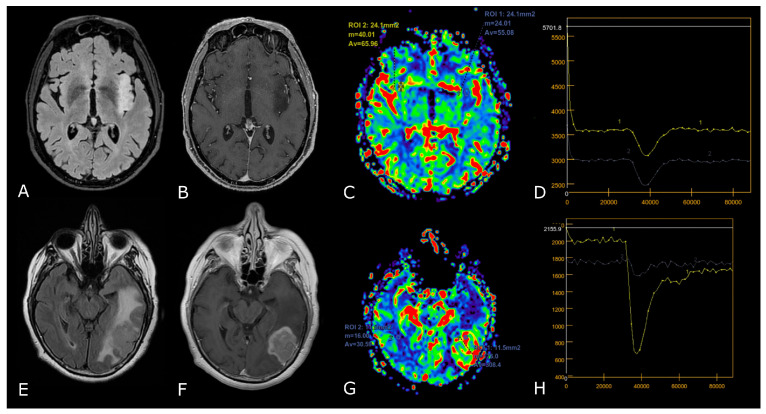
DSC-PWI in differentiating HGG from LGG. Axial T2-FLAIR (**A**) and post-contrast T1w (**B**) of a low grade IDH-mut left insular astrocytoma. DSC-PWI demonstrates normal rCBV (**C**) and complete return to baseline of the signal-intensity-time curve (**D**). The second row shows axial T2-FLAIR (**E**) and post-contrast T1w (**F**) of a left temporal-occipital GB. DSC-PWI demonstrates increased rCBV (**G**) and reduction of PSR consistent with BBB breakdown (**H**).

LGGs are mostly characterized by native vessel while the hypoxic angiogenic microenvironment of HGGs presents with an increased number of leaky vessels [23]. These features are reflected by increased microvascular proliferation and increased BBB permeability in more aggressive tumors. In fact, numerous studies have demonstrated DSC-rCBV and Ktrans to be consistently higher in HGGs. ASL-derived CBF has also been reported to be higher in HGGs [24,25]. K.M. Schmainda et al. reported DSC-rCBV of 1.4 to have sensitivity/specificity of 90/77% in distinguishing LGGs from HGGs [26]. However, several papers suggest different threshold values, so that no clinically useful threshold values are currently available (Figure 4) [27,28].

Of greater clinical importance, it must be remarked that increased DSC-CBV and DCE-Ktrans could be predictive of decreased progression free survival (PFS) and overall survival (OS) in diffuse cerebral gliomas, and that an increase in DSC-CBV during follow-up of LGGs could be an unfavorable prognostic factor as it suggests the possibility of malignant transformation [29,30,31,32].

#### 3.2.2. Differentiating Tumors on the Basis of the Genetic Profiles

After the introduction of the WHO 2021 classification of CNS tumors, PWI has been the subject of numerous studies to non-invasively identify molecular characteristics of primary BTs, in order to actually determine tumor aggressiveness and grading [22].

IDH mutation can regulate hypoxia-inducible factor 1-alpha (HIF-1α), which is a driving force in tumorigenesis and angiogenesis, thus suppressing aggressive behavior (such as angiogenesis) [33]. Consistent with this, mutation of IDH status has been correlated with lower DSC-CBV and DSC-CBF [23], as compared to IDH-wt, whereas PSR is reported to be lower in IDH-wt tumors [23]. DCE studies mainly report that IDH-mut gliomas exhibit decreased Ktrans, Vp, Ve, and AUC [23].

Other relevant molecular mutations concern the EGFR amplification and the TERTp mutation [34]. EGFR amplification is known to accelerate tumor angiogenesis and the induction of proangiogenic factors; increased DSC-CBV, and DCE-Ktrans have been reported in EGFR-amplified GB [23]. Higher expression of TERT was found to be associated with resistance to anti-growth signals and angiogenesis, which could explain the significantly higher mean values of DSC-CBV, CBF and DCE-Vp in TERTp mutant IDH-wt gliomas. Ultimately, O(6)-methylguanine-DNA methyltransferase (MGMT) promoter methylation is known to be associated with an improved response to temozolomide and longer overall survival. MGMT-methylated gliomas exhibit decreased DSC-CBV, whereas unmethylated gliomas show increased Ve and Ktrans [23].

#### 3.2.3. Differentiating Recurrent Tumor from Pseudoprogression and Radiation Necrosis and Be Aware of Pseudoresponse

**Figure 5:**
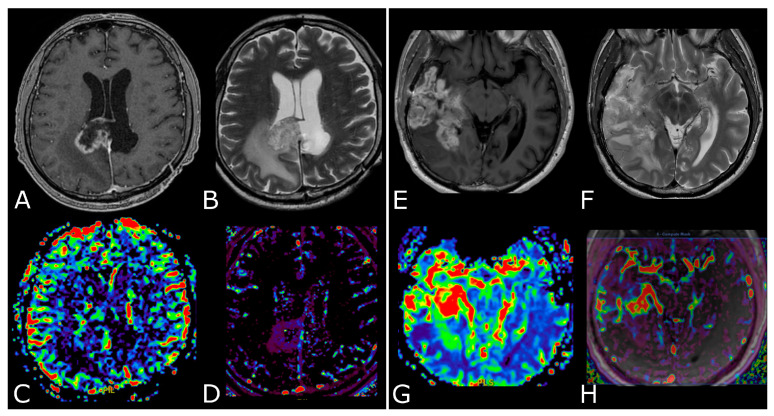
Post-treatment changes vs. disease progression. (**A**–**H**): axial 3D-FSPGR post-contrast T1w images (**A**,**E**) and T2w images (**B**,**F**) with corresponding DSC-CBV (**C**,**D**) and DCE-Ktrans perfusion maps (**G**,**H**) of two IDHwt GBs 1 year after treatment (surgery and radio-chemotherapy). In the left panel the enhancing tissue shows low DSC-CBV and DCE-Ktrans, consistent with post-treatment changes. In right panel the enhancing tissue shows areas of increased DSC-CBV and DCE-Ktrans, suggesting disease progression.

The differences between PsP and radiation necrosis (RN) are related both to the timing of presentation, i.e., 3–6 months for PsP and 1 year after RT in RN, but also to the different pathophysiology whereby RN presents as permanent damage to the brain tissue, necrosis, and vascular thrombosis [35,36,37]. Many DSC studies have shown that CBV is lower in areas of RN or PsP than in those of tumor progression. DCE studies are less numerous but have shown that patients with PsP and RN had significantly lower Ktrans values than patients with tumor progression (Figure 5) [23,29,30,31,35,36,37]. Studies focusing on PsP or RN specifically using ASL are scarce, but one study confirmed that the normalized ASL-CBF ratio was significantly higher in tumor progression than in radiation injury [38].

On the opposite spectrum, favorable false imaging signs are present in the PsR as it occurs with the use of antiangiogenic treatment, i.e., inhibitors of VEGF, that induce the normalization of the cerebral BBB, thus leading to a reduction of the DSC-CBV which, however, represents only a consequence of the alterations of vascular permeability and therefore is not correlated to the efficacy of the treatment [39,40].

## 4. Diffusion Weighted and Diffusion Tensor Imaging

### 4.1. DWI in Brain Tumors: Technical Notes, Clinical Application and Prognosis

Diffusion weighted imaging (DWI) is based on the random thermic motion, or Brownian motion, of water molecules in tissues; “isotropic diffusion” is defined as unhindered diffusion of water molecules, while “anisotropic diffusion” refers to restriction of movement in some directions [41].

DWI enables calculation and mapping of the apparent diffusion coefficient (ADC) of the respective target volume in vivo. Since ADC tumor profiles reflect the corresponding microscopic tissue architecture, DWI has evolved as an important MRI modality, especially in cancer imaging [42]. In fact, ADC can serve as an indirect measurement of cellularity, since in solid tumors, increased cell density will limit free diffusion in the extracellular space, resulting in lower ADC values (Figure 1D) [43]. On the contrary, cystic, and necrotic areas show high ADC values, corresponding to more free diffusion of water molecules in comparison with normal tissue (Figure 2C,D). Similarly, high ADC values are characteristic of peritumoral edema.

DWI has also shown to be useful in glioma grading, in assessing the growth potential of gliomas, and in suggesting clinical prognosis [44].

HGGs show lower ADC compared to LGGs, even in the peritumoral edema, and it is reported that low ADC values correlate with high expression of Ki67 expression, indirectly reflecting proliferation and malignancy [45,46]. Moreover, an ADC cut-off value of 0.0003156 is reported to indicate grade 4 astrocytoma with a sensitivity and specificity of 71% and 100%, respectively [42]. Regarding prognosis, low tumor ADC is reported to be associated with shorter survival times [47,48].

In recent years, a few studies have analyzed the correlations between genetic alterations and diffusion imaging in gliomas. IDH-mut tumors showed significantly higher ADC values compared to the IDH-wt [49,50]. Additionally, Cui et al. reported a threshold mean ADC of 1565.9 × 10^−6^ mm^2^/s to differentiate 1p/19q codeleted WHO grade 2 gliomas from those without codeletion, with 72% sensitivity and 88% specificity [51]. Higher ADC values were also reported to correlate with MGMT promoter methylation in HGGs [52,53], whereas lower ADC values were found in GB with EGFR amplification compared to that without [54].

During follow-up, ADC can be useful for differentiating treatment-related changes (TRCs) from tumor progression in treated HGGs [55]. Through qualitative analysis, Lee et al. found that the occurrence rate of homogeneous or multifocal high signal intensity of tumor progression on DWI is higher than that of PsP, and a mean ADC value lower than 1200 × 10^−6^ mm^2^/s is more common in tumor progression than in PsP [56]. On the contrary, several meta-analyses have demonstrated that diffusion MRI is not suitable for differentiating tumor progression from RN when used alone, and its diagnostic accuracy is the lowest among all advanced MRI techniques [57].

Evaluation of gliomas with DWI has become standard; however, important limitations must be considered when interpreting studies. Hemorrhages, paramagnetic materials, geometric distortion, and susceptibility artifacts can significantly affect the quality of DWI images [58]. Furthermore, HGGs have shown upregulation of aquaporin channels, thereby altering the true estimate of diffusion properties in tumoral and peritumoral environment [59].

### 4.2. Diffusion Tensor Imaging: Technical Notes and Clinical Application

In white matter tracts (WMTs) diffusion is “anisotropic”: higher following the direction of fiber bundles and lower perpendicular to them, due to multiple factors including myelination, axon density and diameter, and axonal membrane integrity [60]. In diffusion tensor imaging (DTI) studies, diffusion is evaluated in multiple different directions and each point of the imaged tissue is represented by “diffusion tensors”, a mathematical model that describes the multidimensional process of diffusion in different axes [61].

**Figure 6:**
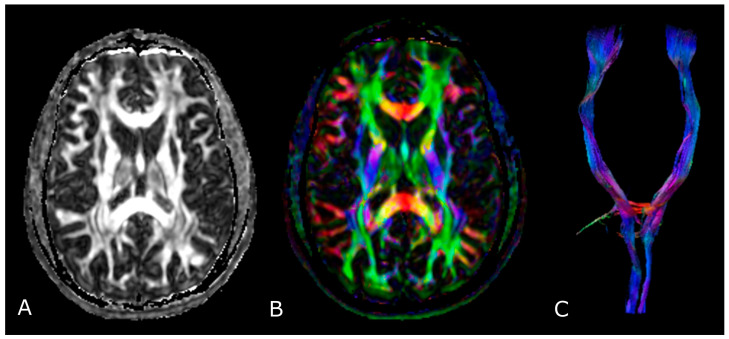
Diffusion Tensor Imaging: (**A**) normal FA map without any directional information; (**B**) Combined FA and directional map. Colors indicate directions as follows: red, left-right; green, anteroposterior; blue, superior-inferior. Brightness is proportional to FA; and (**C**) 3D visualization of normal corticospinal tracts.

The most used indices in DTI are the mean diffusivity (MD), which corresponds to the magnitude of diffusion, and fractional anisotropy (FA), which scales from 0 (completely isotropic) to 1 (completely anisotropic) [60]. DTI images are commonly displayed as color encoded FA maps, with standardized colors for the different fiber orientations: red (left-right), green (antero-posterior), and blue (cranio-caudal) (Figure 6B,C) [60]. With the use of specific software and through the definition of a seed region of interest (ROI), fiber tractography enables 3D visualization of fiber bundles, thus allowing in vivo projection of brain WMTs (Figure 6C).

In the management of brain gliomas, DTI has been applied to different levels: preoperative tissue characterization (glioma grading) [62]; planification of surgical interventions (individuation of ‘functional’ areas to spare) [63,64,65,66]; prediction of post-operative clinical deficits [67,68,69]; intraoperative definition and adjustment of tumor location and extent of resection [63,70,71]; and post-operative assessment, RT/radiosurgery planning and CRT outcome monitoring (recurrent tumor versus RN) [72,73,74].

#### 4.2.1. Tumor Grading and Extension

Some studies evaluated the differences in FA between LGGs and HGGs, with various results [75,76,77]. Usually, in HGGs, the coexistence of fiber destruction/infiltration and the increasing cellular density and vascularity lead to a modest decrease in FA values when compared to LGGs, in which cells are loosely distributed in a fibrillary matrix leading to a significant decrease in FA [78,79].

**Figure 7:**
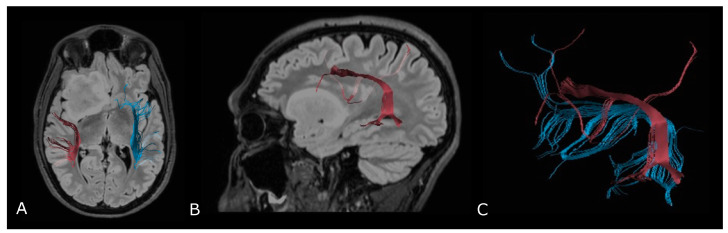
Tractographic reconstruction of arcuate fasciculi. DTI of the direct pathway of both arcuate fasciculi (AF) fused with anatomic axial (**A**); and sagittal (**B**) FLAIR images. The right AF on the affected side is superiorly and posteriorly displaced by an IDH-mut frontal-temporal-insular astrocytoma; (**C**) 3D rendering of both arcuate fasciculi.

Regarding tumor extension, authors reported that white matter surrounding neoplastic brain tissues shows abnormalities in diffusion properties (i.e., reduction in anisotropy) determined by different mechanisms: fiber destruction, fiber dilution (tumor or vasogenic edema spread intact fibers apart), or fiber degradation, thus making it difficult to identify tumor boundaries (Figure 7) [80,81].

#### 4.2.2. Presurgical/Intraoperative Assessment

BT surgery fundamentally aims to achieve both maximal tumor resection and preservation of essential brain functions (i.e., motor, language, and visual function). While direct electrical stimulation (DES) is regarded as the gold standard for mapping brain function, numerous studies have reported the improvement of clinical outcome for glioma patients when DTI-based neuronavigation was used [82]. In fact, by measuring tumor proximity to WMTs, preoperative tractography actually aids in the selection of optimal surgical approaches and in predicting the post-operative functional burden of the resection [83].

However, the so called ‘brain shift’, that refers to the intraoperative modification of anatomical relationships of the cerebral tissue, affects the reliability of preoperative imaging data [84,85], and it is reported to be as large as 20–30 mm at the cortical surface [86]. Thus, DTI results must be interpreted with caution, and a critical safety distance of 5 mm is often used in clinical settings, although many consider it to be arbitrary [87]. Recently, intraoperative DTI has been proposed to limit the impact of such variations in the definition of the extent of resection, thanks to fast imaging acquisition (less than 20 min) and ready integration into navigational systems [70,88,89]; however, cost and availability severely limit current clinical applications.

Finally, recent studies in the literature suggest that a combination of electrophysiological brain mapping with functional navigation (fMRI/Magnetoencephalography data and DTI-based fiber tracking acquired before or during surgery) should be used in order to achieve maximal safety [84,90].

#### 4.2.3. Radiotherapy/Radiosurgery Planning

The utilization of DTI in RT planning has the potential to guide the optimization of radiation beam distribution, and of reducing the dose administered to radiosensitive areas of brain [72]. Several papers have evaluated the integration of DTI-reconstructed corticospinal tract into the planning system [91,92]; more recent research focused on multiple WMTs that are associated with radiation-induced cognitive decline, such as superior longitudinal fasciculus, inferior fronto-occipital fasciculus, and uncinate fasciculus [93,94,95]. In a recent systematic review, the integration of DTI data improved the safety of RT/radiosurgery by sparing essential WMTs without limiting target dose and coverage [72]; only a few studies reported minimal neurologic sequelae by using DTI-guided treatment plans [96].

#### 4.2.4. Differentiation between Recurrent Tumor and Radiation Injury

Usually, after radiation injury, white matter fiber bundles are extensively damaged, with almost no normal fibers and cell structures left. As a consequence, FA values of PsP or RN are considerably much lower than those of tumor progression [57]. Several studies have also pointed out that the FA ratio in RN or PsP was lower than that in recurrent tumors [73,97]. However, another retrospective study showed that DTI metrics were unable to differentiate recurrent tumors and PsP compared to morphologic MRI features. These discordant results might be a mirror of the weaknesses in DTI studies, affected by several factors, either structural (interindividual variability, extracellular volume, tumor density, gliosis/fibrous scar, etc.) and technical (b value, number of directions), which limit their reliability, reproducibility and use in clinical practice [82].

## 5. MR Spectroscopy

### 5.1. Principles of MR Spectroscopy, Metabolites and Their Function

MR spectroscopy (MRS) is a non-invasive in vivo technique that allows for the measurement of biochemical changes in the brain, especially in the presence of BTs. The vast majority of brain MRS studies in vivo use the proton–hydrogen (1H) nucleus, for its abundance and for the higher applicability within standard clinical MRI scanners, whereas interest remains in nuclei such as phosphorus-31(^31^P) and carbon-13 (^13^C), particularly at high magnetic fields and for isotopically labeled and/or hyper-polarized molecules.

The MR spectrum comprises a set of peaks (or resonances) of different metabolites distributed along the x-axis and labelled in parts per million (ppm). The amplitude of the resonances is measured on the y-axis using an arbitrary scale; the area beneath the peak represents the concentration of the metabolite [98,99].

It is possible to use two main methods to select the brain volume to analyze:

1. Single voxel (SV) spectroscopy—provides a spectral trace of metabolites within a voxel (1–8 cm^3^) selected by the operator;
2. Multi Voxel (MV) spectroscopy—also called “MR spectroscopic imaging” (MRSI), or “Chemical Shift Imaging” (CSI)—allows the selection of a large volume of tissue, comprising many voxels (~0.5–1 cm^3^ in volume), each giving rise to a spectrum simultaneously [98,100].

**Figure 8:**
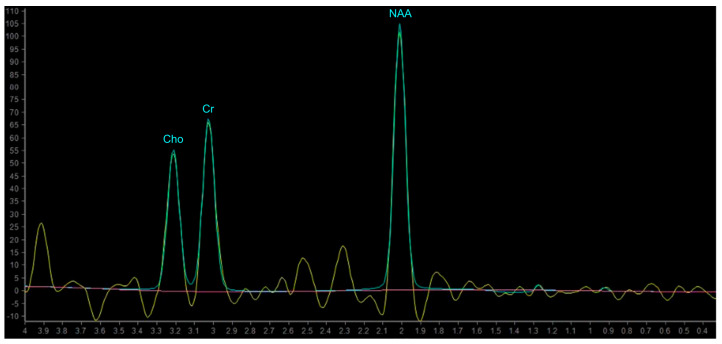
Example of normal spectrum (SV TE 144ms). Normal MR spectrum demonstrating Cho, Cr and NAA peaks.

In proton MRS, the Time of Echo (TE) conditions the number of measurable neurometabolites. At short TE (e.g., TE = 35 ms or less) it is possible to detect the three main peaks normally observed in the brain—choline (Cho), creatine (Cr) and N-acetylaspartate (NAA)—and other compounds which may pathologically increase their concentration, such as myo-inositol (mI), lipids (Lip) and glutamate-glutamin (Glx). Using longer TE (TE = 144 or 280 ms), apart from Cho, Cr and NAA, it is possible to better detect the peaks of molecules with a longer T2, such as lactate (Lac) (Figure 8) [101]. The resonance peaks and biological significance of the major metabolites are listed in Table 3 [98,101,102,103]; unfortunately, there are no unequivocal cutoff metabolite signal ratios that clearly distinguish neoplastic from nonneoplastic conditions. Published MR spectroscopic results showed a sensitivity of 79% and a specificity of 77% for a choline/NAA ratio greater than 1 as an indicator of a neoplastic process [104]; the main ratios are listed in Table 4.

Phosphorus-31 MR Spectroscopy (31P MRS) allows for oinformation about cellular energy or membrane metabolism to be obtained, enabling direct measurement of energy metabolites such as phosphocreatine (PCr), adenosine-triphosphates (ATP), inorganic phosphate (Pi), as well as indirect evaluation of intracellular pH and cell membrane phospholipids composition through phosphomonoesters (PME) and phosphodiesters (PDE) [105]

The PCr/ATP ratio has been described as a marker for the energetic state of a tissue, the PCr/Pi ratio for the oxidative capacity, the Pi/ATP ratio for ATP turnover, the PME/PDE ratio as a surrogate for membrane turnover, and ratios between the membrane-related and the energy-related ratios have been described as a reflection of tumor growth [106].

**Table 4:** Standard metabolic ratios [98,104,107].

	NORMAL	ABNORMAL (Neoplasm)
NAA/Cr	2.0	<1.6
NAA/Cho	1.6	<1.2
Cho/Cr	1.2	>1.5
Cho/NAA	0.7	>1.0

### 5.2. MRS in Brain Tumors

Proton MRS has been widely applied to BTs, helping to differentiate primitive tumors from possible mimics and other tumors, to assess tumor malignancy, and to evaluate therapeutic response. However, because of lesion variability/heterogeneity, overlap between different tumor types and between neoplastic and non-neoplastic lesions, MRS should never be used alone for the diagnosis of a brain lesion but always together with other conventional/morphological and advanced MRI modalities.

#### 5.2.1. Differentiating HGG from LGG

Nearly all BTs show decreased NAA—as a consequence of replacement of neuronal cells by tumoral, necrotic, and reactive tissues, and possibly reduced NAA synthesis, and increased levels of Cho, as a consequence of increased density of proliferating tumor cells [98,100,107].

**Figure 9:**
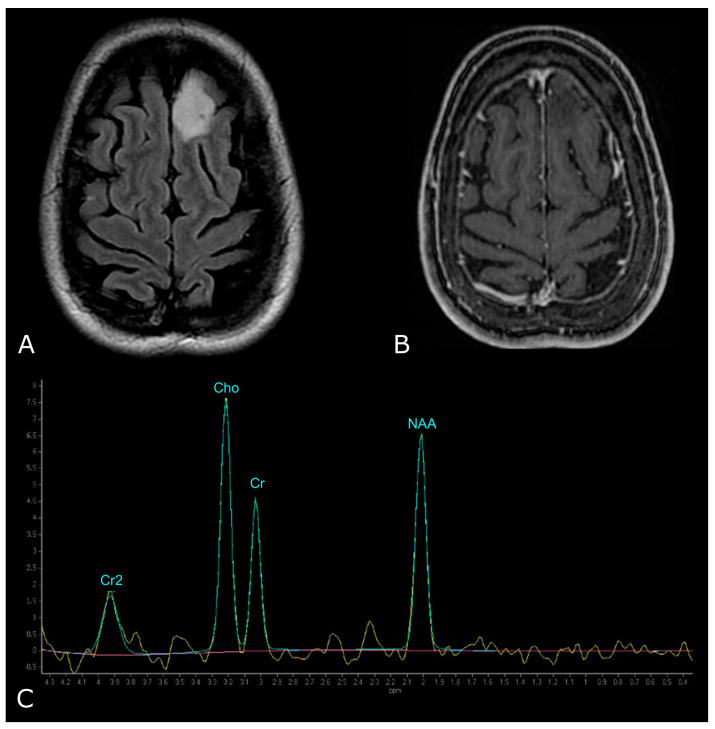
MR Spectrum of LGG (SV TE 144ms). (**A**,**B**) Axial T2-FLAIR and post-contrast T1w of a left frontal low grade IDH-mut and 1p/19q-codeleted OD. (**C**) SV MRS demonstrates moderate Cho elevation and NAA reduction.

However, LGGs usually show either modest Cho elevation or NAA reduction, sometimes accompanied by increased mI and mI/Cr ratio (Figure 9). Moreover, LGGs typically lack Lac and Lip peaks, so that the appearance of Lac and Lip within the tumor is believed to suggest transformation to HGGs.

**Figure 10:**
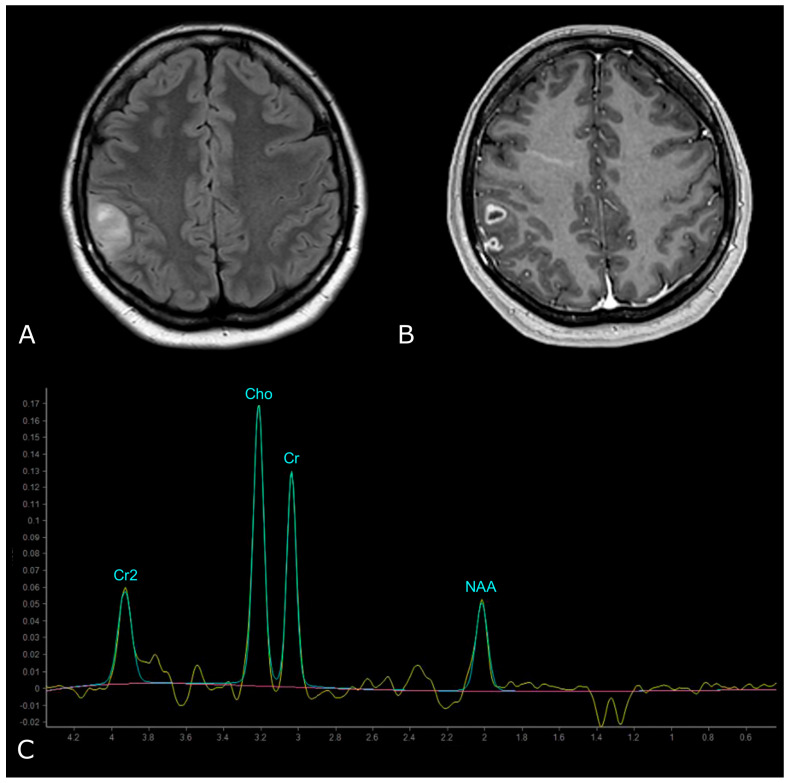
MR Spectrum of HGG (SV TE 144ms). (**A**,**B**) Axial T2-FLAIR and post-contrast T1w of a right parietal HGG, with necrotic areas; (**C**) SV MRS demonstrates a prominent increase in Cho and a decrease in NAA, with high Cho/Cr and low NAA/Cho ratios. Double negative peak of lactates is also present, consistent with the presence of necrosis.

On the other hand, HGGs tend to have more dramatic MRS changes, including a marked increase in Cho and decrease in Cr, NAA, and mI. Thus, higher Cho/Cr and lower NAA/Cho ratios suggest HGGs as opposed to LGGs (Figure 10). To note, pilocytic astrocytomas are reported to have a low NAA/Cho ratio, despite their benign nature, and some authors have reported low Cho levels in HGGs, which may be due to the presence of necrosis [98,100].

Elevated levels of Cho and reduced levels of NAA together can also distinguish regions of tumor from the normal brain beyond the enhancing lesion and can be used to guide tissue biopsy to the most aggressive part of the tumor [108].

Recently, hyperpolarized magnetic resonance techniques (3T scanner) have opened up new opportunities for the metabolic imaging of brain gliomas, allowing the detection of 2HG, a oncometabolite pooled in IDH-mut glioma cells; thus, 2HG MRS may have great potential in clinical practice, representing a noninvasive modality to detect IDH mutation [109].

Regarding 31P MRS, numerous studies demonstrated that an increase in the value of PME is related to malignant progression and increasing grade of tumor malignancy. Typical features of phosphorus spectra of proliferating intracranial tumors are predominant PME peaks and increased values of PDE peaks as a result of the overall metabolism of higher cellular density. Therefore, the PME/PDE ratio can serve as an index of the metabolism of membrane phospholipids and reflect changes in the rate of membrane synthesis or metabolic turnover.

Furthermore, tumor tissue also showed increased values of ATP, as well as Pi levels and, conversely, decreased PCr, demonstrating high energetic demands and mitochondrial inefficiency, leading to an anaerobic metabolic turnover. An increased Pi is associated with a dysfunction of the respiratory chain, which is a hallmark of tissue hypoxia in brain tumors.

Therefore, PME (mostly PME/PDE), followed by PCr/Pi ratios, seems to be the most useful marker for the detection of tumor tissue in 31P MRS ad for evaluating progression to a higher grade [105].

#### 5.2.2. Prediction of Survival and Response to Therapy, Differentiating Recurrent Tumor from Pseudoprogression and Radiation Necrosis

High Cho/NAA and Cho/Cr ratios and the combined high Lac and Lip signal are associated with a higher risk of poor outcome [100,107]. Also, increased Cr was found to be a significant predictor for tumor progression, while gliomas with decreased Cr appeared to have longer progression-free times and delayed malignant transformation [107,109].

A decreased mean tumor Cho/NAA ratio and decreased normalized Cho levels may indicate response to therapy after completion of external beam RT. Moreover, the Lac/NAA ratio in association with the change in Cho/Cr ratio after RT (4 weeks) are predictive of better outcomes [110].

MRS also provides information on tumor heterogeneity, helping to differentiate residual or recurrent tumor from RN on follow-up [100].

MR spectra obtained from regions of recurrent or residual tumors tend to maintain or show an increase in their Cho concentration whereas those areas corresponding to RN tend to have a lower Cho signal, decreased NAA and decreased Cr. RN is also more likely to show elevation in Lip and Lac [98,103].

Several studies have also described differences in energy and membrane metabolism detected with 31P-MRS between stable and progressive disease, given that metabolism of phospholipid cell membrane turnover is one of the major indicators for tumor growth.

In a recent paper, regional differences were shown between contrast-enhancing tumors and normal-appearing brain tissue (adjacent and distant from the enhancing tumor) in patients with GB treated with Stupp regimen; in patients with stable disease, at the FUP scan (1 month) Grams et al. demonstrated significantly higher PCr/ATP and PCr/Pi ratios in contrast-enhancing tumors. In the areas adjacent to the enhancing tumor, an increase in the energetic state (PCr/ATP) and oxidative capacity (PCr/Pi), a decrease in ATP turnover (Pi/ATP) and tumor growth (PME/PCr), as well as normalization of membrane turnover (PME/PDE), in comparison to baseline and healthy controls, occurred under therapy, and was more pronounced in patients with stable disease. In the controlateral hemisphere, an up-regulation of the energetic state (PCr/ATP) was found, which was more marked in patients with SD, demonstrating that cerebral energy and membrane metabolism is modified in the entire brain in patients with GB [106].

**Figure 11:**
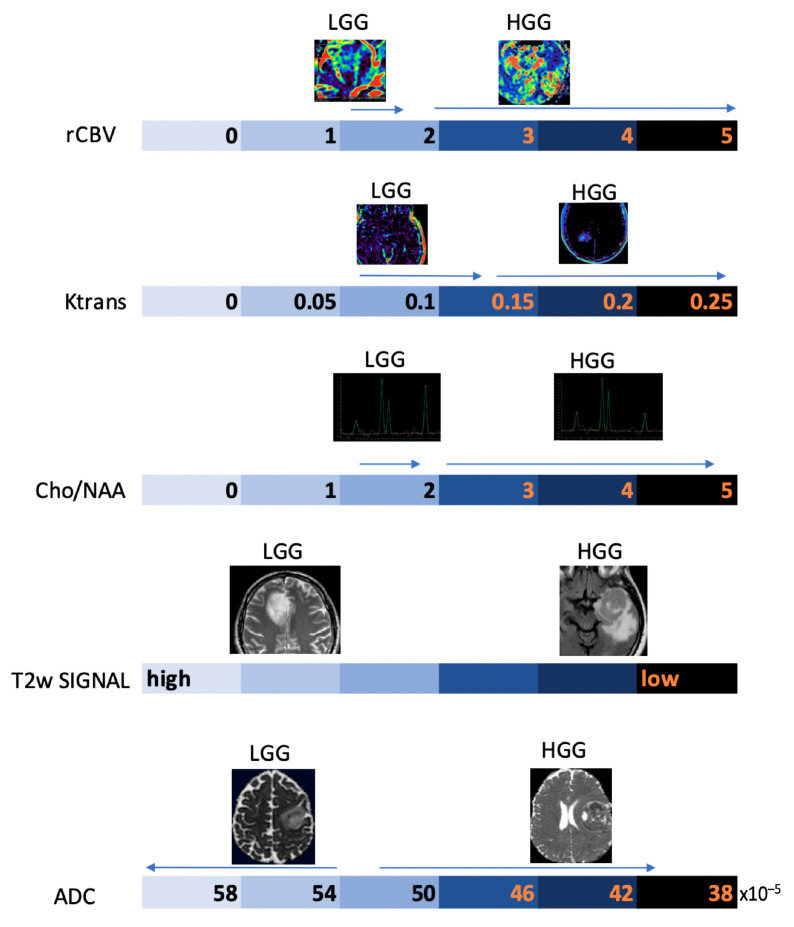
Schematic representation of the main MRI parameters used in the diagnostic work-up of gliomas on an arbitrary numerical scale, with indication of the main values associated with the degree of biological aggressiveness.

Below, we propose a practical overview of the main morphological and non-morphological MRI parameters useful in the daily work-up of patients with primary BTs (Figure 11).

## 6. Functional MRI

Functional MRI (fMRI) measures brain activity by detecting changes associated with blood flow and relies on blood-oxygen-level-dependent (BOLD) contrast. Specifically, BOLD contrast is based on the different ferromagnetic properties of oxygenated (diamagnetic, oxHB) and deoxygenated (paramagnetic, dHB) hemoglobin. As a result of a stimulus involving neuronal activation, there is a greater consumption of oxygen in this area and consequently a localized increase in dHB. To cope with oxygen demand, there is a subsequent increase in oxHB concentration and dHB elimination, resulting in a reduction in the area inhomogeneity in T2*, and consequently, an increase in the BOLD signal in that area [111].

To date, the use of fMRI is targeted towards pre-surgical planning and identification of eloquent areas (language [112,113], motor [114], vision and memory areas [115]) adjacent to the tumor, where the need for an extensive resection must be counterbalanced by the sparing of functional cortical and subcortical structures. The assessment of lesion-to-activation distance has been considered relevant for the evaluation of postoperative outcomes. In general, it is assumed that the risk of postoperative loss of function, assessed with fMRI, is significantly lower when the distance between tumor periphery and BOLD activity is at least 10 mm [116,117].

**Figure 12:**
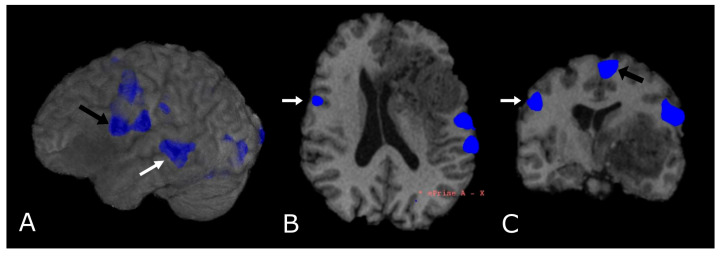
Language task-based fMRI. fMRI correlation maps of cortical activation during language tasks in a patient with frontal-temporal-insular GB. Blue represents areas of increased cortical activation. (**A**) 3D surface rendering with BOLD signal overlay reveals the activation of Broca’s Area, displaced posteriorly by the lesion (black arrow; expressive speech) and in the superior temporal gyrus (white arrow, Wernike’s Area, receptive speech); (**B**,**C**) Axial and coronal deskulled T1w-BRAVO with BOLD signal overlay confirm the activation and the dislocation of Broca’s Area and shows a significant but smaller activation in the right inferior frontal gyrus (white arrow’s head). Black arrow’s head indicates the supplementary Motor Area (**C**).

Most fMRI clinical exams are task-based, so that the subject is asked to perform an action in response to a stimulus aimed at the activation of a specific cortical area, alternated with control/rest phases. Alterations of the BOLD signal are then analyzed to localize the area under consideration (Figure 12). Task-based fMRI has proven to be a valid and highly sensitive tool for localizing the distinct eloquent cortical and subcortical areas before surgery in glioma patients, also showing good accuracy when compared to intraoperative stimulation mapping data. Task-based fMRI is reported to be more accurate in LGGs than in HGGs, due to neurovascular uncoupling [118,119,120], the presence of arteriovenous shunting, mechanical vasoconstriction caused by tumoral mass effect, and the presence of intratumoral hemorrhages.

In recent years, fMRI of the resting state network (rs-fMRI) has attracted considerable attention; it consists of the low frequency spontaneous hemodynamic fluctuations during rest, to investigate the functional architecture of the brain [121]. Rs-fMRI was introduced by Biswal and his group in 1995, when they found out that resting state signals are consistent low frequency fluctuations in the range 0.01–0.08Hz [122]. Although the use of rs-fMRI is mainly confined to research purposes, recent studies have shown similar reliability compared to task-based fMRI with respect to mapping the sensorimotor network in healthy subjects [123,124]; thus, rs-fMRI has the potential to become the noninvasive standard of care for surgical planning and prognosis [121,125,126].

Finally, it must be mentioned that fMRI can be used as a guide for DTI tractography [127]. In fact, pre-surgical planning integrating fMRI and DTI may help to identify, and, subsequently, allow surgeons to avoid, areas of important functional and anatomical redistribution.

## 7. Radiomics

Radiomics refers to the use of a series of characteristics (features) of an area, called region of interest (ROI), which can be delineated either manually or automatically, within a radiological image. Features are numerical indicators that describe the properties of ROI regarding gray gradations, texture (or graininess), the presence of patterns (particular configurations or structures). The goal of radiomics analysis is mainly to make the interpretation of the image more objective and contribute significantly to the visual potential of radiologists, helping in the identification of patterns present in the image but not detectable to the naked eye. Radiomics features are divided into first order and second order. First order features include mean, median, standard deviation of Hounsfield values, entropy, indicating the degree of unpredictability of gray level distribution, skewness, indicating histogram symmetry, and Kurtosis. Among the main second-order features there are descriptors of the contrast group, type dissimilarity and homogeneity, descriptors related to order, type angular momentum, energy and entropy, statistical descriptors that analyze the frequencies of pairs of values, average type, standard deviation and correlation, descriptors that analyze the differences in gray levels between each element of the image and those immediately adjacent, such as coarseness, contrast, and activity [128].

**Figure 13:**
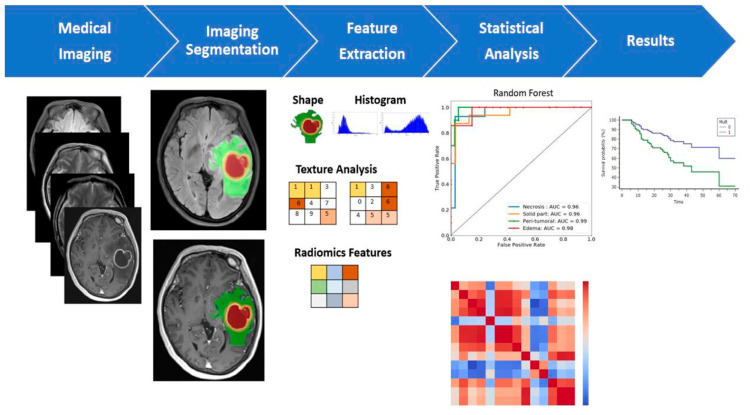
Multiparametric MRI-based radiomic analysis. A multiparametric MRI-based radiomic analysis in steps: (1) medical imaging acquisition, (2) imaging segmentation, (3) feature extraction, (4) statistical analysis, and (5) results. The tumor ROI on all MR slices to extract the radiomic features. Features such as tumor shape, histogram, and texture features were extracted from the ROIs to discriminate the biological processes of GB habitats and facilitate personalized precision medicine [138].

Following the extraction of the image-derived features, it is possible to process them to make correlations to specific outcomes using statistical models or machine learning. Statistical models are used to determine mathematical relationships between variables and outcomes; the most used are univariate and multivariate analysis. Machine learning models consist of a system which does not need programmed instructions to work, since it is capable of learning from the data itself; these models are usually considered more reliable than statistical ones when it comes to predictive purposes because they rely on fewer mathematical assumptions, minimal human error, and they are built from larger and more detailed datasets (Figure 13).

**Figure 14:**
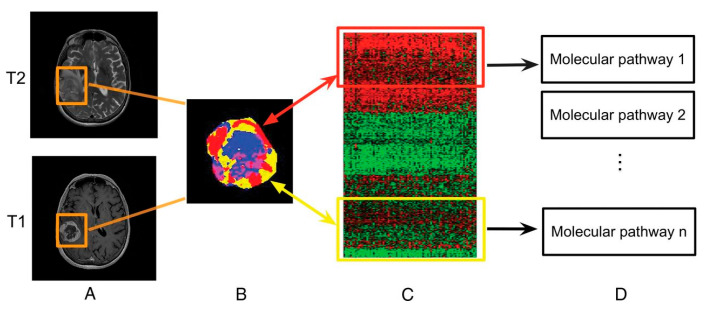
Linking subregional imaging to molecular profiles in GB. In this example, tumor subregions (**B**) are defined by jointly clustering on contrast-enhanced T1WI and T2WI (**A**). These subregions correspond to red (high T1WI and high T2WI), yellow (high T1WI and low T2WI), blue (low T1WI and high T2WI), and pink (low T1WI and low T2WI) areas. The defined tumor subregions enable quantitative spatial characterization, offering a means to noninvasively assess specific molecular activities (**C**) with enriched molecular pathways (**D**) [139].

Radiomics is a fast-growing field in radiology where images from conventional radiological examinations are converted into extractable quantitative data that can then be used to decode the tumor phenotype for applications ranging from improvement of the diagnosis to the prognosis, to the prediction of the response to treatment [129]. In that field, radiogenomics refers to a specific application where imaging features are linked to genomic profiles (Figure 14) [130].

Among clinical applications, radiomics could be useful in the evaluation of mutations in primary tumors, particularly glial tumors.

Lu at al. built a multilevel quantitative imaging model based on post-contrast T1w, T2-FLAIR, T2w, DWI, and ADC to recognize IDH and 1p/19q genotypes of gliomas, with an accuracy up to 89.2% [131]. Radiomics predictors were also built for genetic features implicated in prognosis such as MGMT methylation and EGFRA289V mutations in high grade gliomas, and ATRX mutations in LGGs [132,133,134]. Additionally, Wang et al. built a multidimensional quantitative radiomics model which integrates clinical data (individual patient characteristics and glioma grading), MRI and PET imaging for the differential diagnosis between post-surgical disease recurrence and RN [135].

Radiomics and radiogenomics could be useful in predicting response to RT treatment in combination with temozolomide since the response is strictly dependent on biological heterogeneity and patients would benefit from a therapy with a personalized dose [136].

Although radiomics is continuously expanding, it suffers from several limitations due mainly to the standardization of data, which limits its robustness, reproducibility, and generalizability. In fact, current standards lack validation of results and are characterized by incomplete result reports and unidentified confounding variables in the source database, especially for retrospective data. Moreover, segmentation is used in most imaging studies to ensure high accuracy and fine delineation of irregular tumor margins, but it suffers from subjective factors that make it poorly repeatable [137].

## 8. Artificial Intelligence

**Figure 15:**
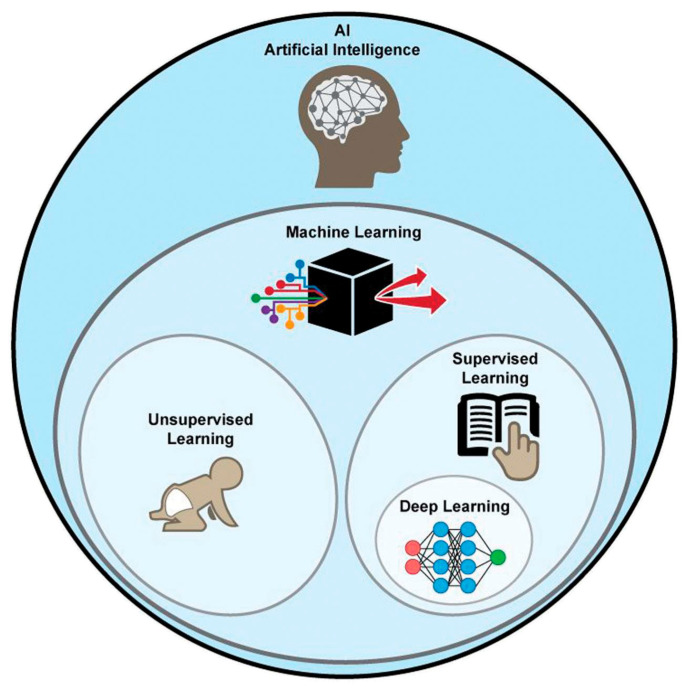
Graphical representation of AI methods. ML is a form of AI, divided into supervised and unsupervised learning. DL is a form of ML, usually based on supervised learning [160].

Nowadays artificial intelligence (AI) is widespread in many fields, such as in the economy, the automotive industry, and medicine. Machine learning (ML) is a type of AI that has the ability to acquire its own knowledge by extracting patterns from raw data. Machine learning methods can be divided into supervised learning, in which the dataset contains features that are used to train the algorithm, and unsupervised learning, in which the algorithm experiences a dataset without pre-existing specifications. Deep learning (DL) is a branch of ML inspired by the way in which the brain processes information. In fact, it is constituted of layers of “artificial neurons” called units, which act in parallel with other units of the same layer and in series with other layers to analyze data. There is a hierarchy of features elaborated by each layer: deeper layers define higher-level features and lower-level features help define higher-level features. The final layer, the so-called output layer, produces the ultimate result (Figure 15) [140,141].

With regard to images acquisition/post-processing, AI can provide many advantages: (1) it produces good quality images from under-sampled data, which means shorter acquisition time, particularly useful with claustrophobic/uncooperative patients [142]; (2) it recognizes and removes motion artifacts [143] and corrects other sources of degradation, such as a low signal to noise ratio [144], magnetic field inhomogeneities and improper water or lipid suppression [142]; (3) it reconstructs full-dose post-contrast images from a low-dose or zero-dose acquisition, fundamental in settings in which GBCA cannot be administered, such as renal insufficiency or pregnancy (but missing lesions <10 mm is reported) [145,146,147]; and (4) it applies algorithms that allow to synthesize images with higher spatial and contrast resolution than originals [148].

Dealing with BTs, AI algorithms allow lesion segmentation, based on one or multiple sequences (the most used are T1w, post-contrast T1w, T2w and FLAIR). AI segmentation divides the brain into different areas such as normal brain tissue, active brain tissue, necrosis, and edema, supporting an early definition of the neurosurgical planning, which is particularly helpful for those tumors which are hard to distinguish from normal tissue due to infiltration and/or unclear boundaries (i.e., GB) [149,150,151,152]. Although AI automation provides benefits such as elimination of inter-observer variability and reduction of inference time, it still has some limits: (1) poor performance of segmentation algorithms if large-scale training datasets are lacking; (2) the necessity of a huge amount of computational and memory resources; and (3) lower performance in cases of poor spatial resolution, ill-defined boundaries, measurement noise, variability of object shapes [149,152,153].

Most of the studies concerning AI application to BTs classification deal with the use of DL algorithms to differentiate four main tumor types: gliomas, metastases, meningiomas, nerve sheath tumors [152]. Their accuracy varies from 70% and 97%, according to the number of tumors included, which is very promising in the perspective of a non-invasive diagnosis, which is safer and “wider” than biopsy. However, recent papers report greater accuracy (higher than 80%) even in distinguishing IDH-mut codeleted 1p/19q tumors, MGMT methylated tumors, and H3-K27M mutation status, a remarkable goal considering their impact on the course of treatment and prognosis [139,154,155,156,157]. Intriguing data obtained by Gao et al. showed that neuroradiologists’ accuracy increased when they had access to DL results and made the necessary modifications and corrections to their diagnoses [158].

AI has also been applied to the evaluation of prognosis and/or identification of tumor progression.

**Figure 16:**
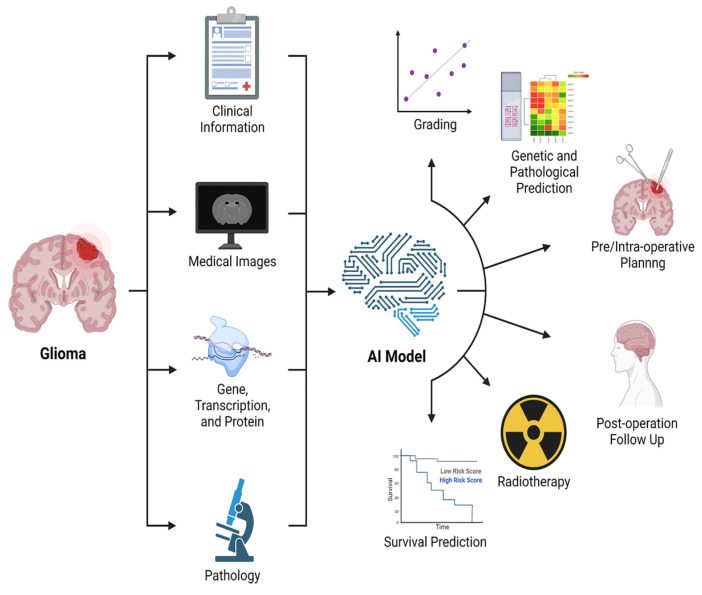
Graphical representation of the main applications of AI in BTs [156].

In fact, several studies have shown that data from DTI and fMRI, analyzed by AI algorithms, are more effective in dividing GB patients into short and long survival groups than histopathologic information alone [159]. Moreover, DL algorithms demonstrated an accuracy of 90% in differentiating between PsP and true tumor progression [156]. Ultimately, DL could help identify molecular/genetic changes occurring during follow-up which could need or address therapeutic changes (Figure 16) [157].

## 9. Quantitative MRI

Longitudinal qualitative comparison of MR images acquired at different sites is limited due to the dependence of signal intensity on magnetic field strength and its inhomogeneities (B0), radiofrequency coil (B1) and imaging parameters (i.e., TE, TR, and flip angle) [161,162]. Moreover, BT pre- or post-treatment evaluation is difficult due to subtle changes in tissue composition not visible on conventional MRI scans [162,163].

Quantitative MRI (qMRI) is a method that isolates the contributions of individual MR contrast mechanisms (T1, T2, T2*) and provides maps (such as the subtraction between pre- and post-contrast T1 or between following exams), which are independent of the MR protocol and have a physical interpretation often expressed in absolute units [161]. Measurement of relaxation times needs a large set of images, each weighted slightly differently, to obtain an adequate sampling of signal evolution and an accurate measurement. Inhomogeneities of B0 or B1 are averaged out by the measurement series, or they are directly measured and corrected thereafter [161,162].

**Figure 17:**
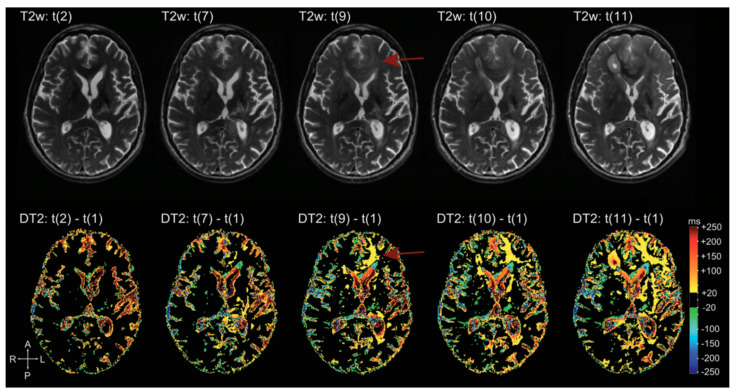
Example of T2 subtraction maps at different time points during follow-up. Upper row: T2 images at different time points. Lower row: T2 subtraction maps (with reference to time point one, not showed) at the same time points. T2 subtraction maps sharpen the evolution of T2 signal during time, hardly visible at the beginning on conventional T2 images (difference more evident at time point 9, red arrow) [168].

There are many applications of qMRI in the evaluation of brain tumors. First, it is used to detect tumor infiltration beyond the enhancing part of HGG, according to the fact that recurrence often arises from resection margins, suggesting that this area represents a non-enhancing infiltration zone. qT1 difference maps (post-contrast T1w—pre-contrast T1w) show moderate GBCA accumulation in peritumoral edema, and in some cases even beyond, not evident in conventional images, signs of subtle BBB leakage, and therefore tumor infiltration [163,164]. Secondly, it allows neuropathological characterization of brain microstructure. In general, BTs show increased T1 and T2 relaxation times. T1, T2, T2* relaxation times are correlated with lactate dehydrogenase, inversely correlated with vessel density; the relative difference between T1 relaxation times pre-to-post GBCA shows a weak correlation with Ki67 and a negative correlation with the amount of necrosis [165]. Third, it is helpful to distinguish between recurrence and post-treatment changes during follow-up. Many studies evaluated patients under anti-angiogenic therapy (e.g., Bevacizumab) and demonstrated that “longitudinal” subtraction maps (particularly post-contrast T1) lead to an earlier detection of recurrence than conventional MRI (Figure 17). Moreover, qMRI aids prediction of OS and PFS: Ellingson et al. suggested that patients with a post Bevacizumab T2 relaxation time higher than 160 ms would progress earlier (both OS and PFS were statistically significant) [166]; Ellingson et al. demonstrated also that post-contrast T1 subtraction maps allow for the prediction of OS and PFS from the decreasing tumor volume after Bevacizumab, with a direct correlation between decreasing volume and OS and PFS (data not showed from conventional MRI) [167].

## 10. Conclusions

Through this review article, we have highlighted the current role of morphological and non-morphological MR techniques in the clinical setting of adult patients with primary BTs.

MRI is the mainstay of modern neuroimaging and nowadays it permits superior structural characterization capturing the cellular, vascular, metabolic, and functional properties of BTs.

The combined use of all MRI techniques is actually the key to reaching the best clinical patient care, independently from the stage of the patient’s disease.

## Figures and Tables

**Table 1:** Summary of the RANO Response Criteria.

Criterion	CR	PR	SD	PD
T1 gadolinium enhancing disease	none	≥50%	<50% but <25% ↑	≥25% ↑ ^a^
T2/FLAIR	stable or ↓	stable or ↓	stable or ↓	↑ ^a^
New lesions	none	None	None	present ^a^
Corticosteroids	none	stable or ↓	stable or ↓	NA ^b^
Clinical status	stable or ↑	stable or ↑	stable or ↑	↓ ^a^
Requirement for response	all	All	All	any ^a^

Abbreviations: RANO, Response Assessment in Neuro-Oncology; CR, complete response; PR, partial response; SD, stable disease; PD, progressive disease; FLAIR, Fluid-attenuated inversion recovery; NA, not applicable. ^a^ Progression occurs when this criterion is present. ^b^ Increase in corticosteroids alone will not be taken into account in determining progression in absence of persistent clinical deterioration [15].

**Table 2:** Main perfusion techniques parameters in brain tumors.

Parameter	Meaning	Units	PWI Technique
CBV	Cerebral Blood Volume	mL of blood/100 g tissue	DSC
CBF	Cerebral Blood Flow	mL of blood/100 g of tissue/min	DSC, ASL
Ktrans	Volume transfer constant between blood plasma and extravascular extracellular space	1/min	DCE
Ve	Extravascular extracellular volume fraction	mL/100 mL	DCE
Vp	Blood plasma fractional volume	mL/100 mL	DCE
AUC	Area under the signal intensity/time curve	mM/s	DCE
PSR	Percentage of signal intensity recovered at the end of the first pass of GBCA, relative to baseline	%	DSC

**Table 3:** Proton MRS Metabolites, peaks and biological significance.

Metabolites	Peaks	Biological Significance
N-Acetylaspartate (NAA)	2.02 ppm	brain-specific molecule, marker for viable neurons (“neuronal marker”)
Choline (Cho)	3.20 ppm	marker of tumor cell proliferation
Creatine (Cr)	3.03 and 3.9 ppm	marker of energetic systems and intracellular metabolism
Lactate (Lac)	doublet peak at 1.33 ppm, inverted below the baseline at long-intermediate TE	marker of anaerobic metabolism
Lipids (Lip)	From 0.90 ppm to 1.30 ppm	marker of cellular breakdown or necrosis
Myo-Inositol (mI)	3.50–3.60 ppm	glial marker
“Glx”: overlapped peaks of Glutamine (Gln) and Glutamate (Glu)	between 2.12–2.35 ppm and 3.74–3.75 ppm	Glutamate: neurotransmitter Glutamine: astrocyte marker
2-Hydroxyglutarate (2-HG)	2.25 ppm	Oncometabolite pooled in tumors with IDH-mut

ppm: parts per million.

## Data Availability

Not applicable.
